# Mechanism underlying Müller cell pyroptosis and its role in the development of proliferative vitreoretinopathy

**DOI:** 10.1016/j.clinsp.2023.100241

**Published:** 2023-07-05

**Authors:** Yue Bai, Maosong Xie, Yihua Zhu

**Affiliations:** Department of Ophthalmology, The First Affiliated Hospital of Fujian Medical University, Fuzhou, China

**Keywords:** Müller cells, Pyroptosis, Proliferative vitreoretinopathy

## Abstract

**Objectives:**

To explore the mechanism underlying Müller Cell Pyroptosis (MCP) and its role in the development of Proliferative Vitreoretinopathy (PVR).

**Method:**

The expression of pyroptosis-related factors, namely, cysteinyl aspartate-specific proteinase (caspase-1), interleukin (IL)-1β, IL-18, and Gasdermin D (GSDMD), was detected by quantitative Reverse Transcription Polymerase Chain Reaction (qRT-PCR) and western blotting at the mRNA and protein levels, respectively, in retinal tissues. Müller and spontaneously Arising Retinal Pigment Epithelia (ARPE)-19 primary cells with GSDMD overexpression or knockdown were cultivated. Western blotting was used to detect the levels of the following pyroptosis-related factors in retinal tissues: caspase-1, IL-1β, IL-18, and GSDMD. Through Cell Adhesion (CA) experiments, the changes in ARPE-19 CA in each group were observed. The migration and invasion of ARPE-19 cells were measured using the Transwell assay. The proliferation of ARPE-19 cells was measured with a Cell Counting Kit 8 (CCK-8) assay. Finally, the expression of the cytokines IL-1β and IL-18 in the ARPE-19 cell culture medium was detected using the Enzyme-Linked Immunosorbent Assay (ELISA).

**Results:**

Compared with the surrounding normal tissues, the expression of caspase-1, IL-1β, IL-18, and GSDMD at the protein and mRNA levels in the retinal proliferative membrane samples of the patients decreased significantly (p < 0.05). MCP significantly enhanced ARPE-19 CA, migration and invasion, proliferation, and cytokine expression (p < 0.05).

**Conclusions:**

MCP can promote the development of PVR lesions.

## Highlights

• MCP enhanced ARPE-19 CA, proliferation, migration, invasion, and cytokine expression.
• MCP can promote the further development of PVR.
• The inhibition of MCP may be the key to the treatment of PVR.

## Introduction

Proliferative Vitreoretinopathy (PVR) is a serious eye disease that can cause blindness.^1^ Since the pathogenesis of PVR has not yet been elucidated, surgery is currently the first-line treatment. However, surgery cannot entirely remove the tightly adhered, thick, and stiff hyperplastic membrane, and retinal rupture can easily occur when tearing this membrane.^2^ Therefore, further exploration of PVR pathogenesis may markedly improve PVR treatment.

Pyroptosis is a recently elucidated type of cell death; it is a pro-inflammatory form of cell death dependent on the expression of Interleukin (IL)-1β, IL-18, and High-Mobility Group protein 1 (HMGB-1).^3^^,^^4^ Morphologically, a pyroptotic cell expands causing a spherical vesicle to form around the nucleus, rupturing the plasma membrane and then immediately resealing it again. As pyroptotic cells expand, the nucleus becomes rounded and shrinks.^5^ Activated cysteinyl aspartate-specific proteinase (caspase-1) can cause pyroptosis, which is characterized by cell lysis and the release of cytoplasmic contents into the extracellular space.^6^ Gasdermin D (GSDMD), a member of the gasdermin family, is a common substrate of caspase-1 and caspase-4/5/11 and an important component involved in pyroptosis.^7^

Recent studies have found that the main components of the PVR membrane are Retinal Pigment Epithelium (RPE) cells, Müller cells (MCs), and the extracellular matrix. The migration, proliferation, and epithelial-mesenchymal transition of RPE cells are closely related to the occurrence and progression of PVR.^8^ Müllercell Pyroptosis (MCP) can promote the progression of proliferative diabetic retinopathy.^9^ However, to date, no studies have reported the role of MCP in the occurrence and progression of PVR.^10^

The present study aimed to elucidate the effects of MCP on RPE cells and determine if it contributes to the occurrence and progression of PVR. To this goal, the authors examined the specific role of MCP in PVR through *in vitro* and *in vivo* analyses. The present findings provide novel ideas for PVR research and treatment.

## Materials and methods

### Patients and specimens

This study protocol was reviewed and approved by The First Affiliated Hospital of Fujian Medical University (Approval n°: MRCTA, ECFAH of FMU [2019]131). All procedures of the study followed ARRIVE guidelines. The authors collected retinal proliferative membrane samples from patients with PVR who received treatment at the hospital from January 2019 to December 2020. The PVR patients the authors included complied with strict inclusion and exclusion criteria.

Inclusion criteria included: 1) Patients with rhegmatogenous retinal detachment complicated by PVR or PVR following retinal detachment surgery and requiring vitreous surgery, and 2) Patients who agreed to participate in this study and completed an informed consent form.

Exclusion criteria were: 1) Patients with diabetes, 2) Other eye diseases, and 3) Combined systemic diseases.

The retinal proliferative membranes were carefully obtained during standard 23 gauge (23G) vitreous body resection with microscopic peeling forceps, immediately placed in a 4% paraformaldehyde solution and incubated in the solution at 4°C overnight.

### Cell culture

Both MCs and ARPE-19 primary cells were purchased from the Type Culture Center of the Chinese Academy of Sciences (Shanghai, China). MCs and ARPE-19 cell lines were cultured in Dulbecco's Modified Eagle Medium (DMEM)/F12 (Life Technologies, Inc., Gaithersburg, MD, USA) supplemented with 10% fetal bovine serum (FBS), 100 mg/mL streptomycin, and 100 U/mL penicillin (Gibco; Thermo Fisher Scientific, Inc., Waltham, MA, USA). All cells were maintained at 37°C in a humidified incubator containing 5% CO_2_.

When the bottom of the flask was approximately 90% filled, the passaged cells were washed first with sterile Phosphate Buffered Saline (PBS), digested with 0.25% trypsin (containing 0.02% Ethylenediaminetetraacetic acid EDTA]), and again passaged 1:3.

### Plasmid acquisition and transfection

The *GSDMD* overexpression plasmid, pc-GSDMD, and the empty plasmid, pcDNA3.1, were constructed by the Chinese Academy of Sciences (Changchun). Specific small interfering (si) RNA (siGSDMD) and Negative Control (si-NC) vectors targeting the expression of GSDMD were synthesized by Wuhan Genesil Biotechnology Co., Ltd. (Wuhan, China.)

MCs were seeded onto six-well plates overnight before transfection. Following the manufacturer's instructions, all oligonucleotides and plasmids were transfected into MCs using Lipofectamine 2000. Transfection efficiency was evaluated using Quantitative Reverse Transcription Polymerase Chain Reaction (qRT-PCR).

### Cell grouping

The analyzed cells were mainly divided into the following groups: RPE cell-only culture group (RPE), RPE and MC co-culture group (RPE+M), RPE and empty plasmid MC co-culture group (RPE+M+pcDNA3.1-NC), RPE and *GSDMD*-overexpressing MC co-culture group (RPE+M+pcDNA3.1-GSDMD), RPE and si-NC-treated MC co-culture group (RPE+M+si-NC), and RPE cells co-cultured with siGSDMD-treated MCs (RPE+M+si-GSDMD).

### qRT-PCR

Total RNA was extracted from tissues and cells using TRIzol reagent (Invitrogen), following which reverse transcription was performed using a Moloney Murine Leukemia Virus Reverse Transcriptase (M-MLVRT) kit (Promega) following the manufacturer's instructions. Specific primers (GeneCopoeia) were used to detect GSDMD and Complementary Deoxyribonucleic Acid (cDNA), and were amplified using SYBR®GreenPCRMasterMix (Toyobo, Osaka, Japan). Quantitative PCR was performed using the SYBR Premix Ex Taq ™II kit (TaKaRa, Dalian, China), following the manufacturer's instructions. U6 small nuclear RNA was used to normalize *GSDMD* mRNA expression. All data were analyzed using the 2^−ΔΔCt^ method.

### Western blot analysis

The cultured cells were lysed with radioimmunoprecipitation assay lysis buffer (Beyotime Institute of Biotechnology, China). Protein concentration was determined using a bicinchoninic acid analysis kit (Pierce). The proteins were then separated using 8% sodium dodecyl sulfate-polyacrylamide gel electrophoresis and transferred to a polyvinylidene fluoride membrane (Roche, Basel, Switzerland). After blocking with 5% skimmed milk at room temperature for 1.5h, the membrane was incubated with the primary antibody overnight at 4°C followed by incubation with the corresponding secondary antibody for 1.5h. The membrane was treated with an enhanced chemiluminescence reagent (Millipore), and the bands obtained were quantitatively analyzed using ImageJ software (NIH, USA). All primary antibodies were purchased from Abcam (Cambridge, UK).

### Cell adhesion (CA) test

The authors coated a 24-well plate with 20 μg/mL fibronectin solution and incubated it overnight at 4°C. Next, the authors inoculated the wells with the prepared single-cell suspension of each group of cells (concentration of 5 × 10^4^/mL) and experimental medium at 37°C, incubated the plate in a 5% CO_2_ incubator for 30 min, washed off the non-adherent cells with PBS, fixed the remaining cells in 4% paraformaldehyde at room temperature for 15 min, and again incubated the cells with 1:1000 Hoechst stain at room temperature for 10 min. The images were observed under a fluorescence microscope, and ImageJ was used to count the number of adherent cells.

### Cell counting kit 8 (CCK-8)

After treatment, the ARPE-19 cells were cultured for 24h. Cells were collected and suspended in a cell suspension. The cells were seeded onto a 96-well plate at a density of 1 × 10^3^ cells/well. For Group 3, the authors replicated the wells and incubated them at 37°C in 5% CO_2_. After incubating for one, two, three, and four days, 10 μL of CCK-8 reagent was added, and the absorbance value of each well was measured at 450 nm using a microplate reader after two hours. Finally, the cell survival rate was calculated using the following formula: cell survival rate% = (experimental group A−solvent A group)/(control group A−solvent A group) × 100%.

### In vitro migration and invasion analysis

To evaluate the changes in the migration and invasion abilities of ARPE-19 cells, the cells were cultured for 24h, following which they were collected and prepared as a cell suspension. The cells were seeded onto 96-well plates at a density of 1 × 10^3^ cells/well. For Transwell migration and invasion measurements, each group of the plates had three replicates. The Transwell chamber (Corning, NY, USA) consisted of a 24-well plate with a polycarbonate membrane having a pore size of 8 μm. To perform migration assays, 2 × 10^4^ cells in 200 μL of serum-free medium were seeded onto the upper chamber, and the lower chamber was filled with 600 μL of medium containing 10% FBS. After 24h, the cells were fixed in 4% paraformaldehyde and stained with 0.1% crystal violet. The number of penetrating cells was counted in six random fields of view in each chamber, following which the average value was calculated. The experimental procedure of the Transwell invasion assay was the same as that of the Transwell migration assay, with the exception of the process of pre-coating Matrigel (BD Biosciences, Bedford, MA) in the Transwell chamber.

### Enzyme-linked immunosorbent assay (ELISA)

After treatment, ARPE-19 cells from each group were cultured for 24h, the cell culture medium was collected, and IL-1β and IL-18 levels in the cell culture liquid of each group were determined using an ELISA kit, according to the instructions of the manufacturer.

### Statistical analysis

All data were analyzed using SPSS version 22.0. The measurement data are presented as the mean ± Standard Deviation (SD). For data conforming to a normal distribution with uniform variance, the difference between the two groups was analyzed using a *t*-test, and the differences between multiple groups were analyzed by one-way analysis of variance. Statistical significance was set at p < 0.05.

## Results

### Expression of pyroptosis-related genes in PVR patients

**Fig. 1:**
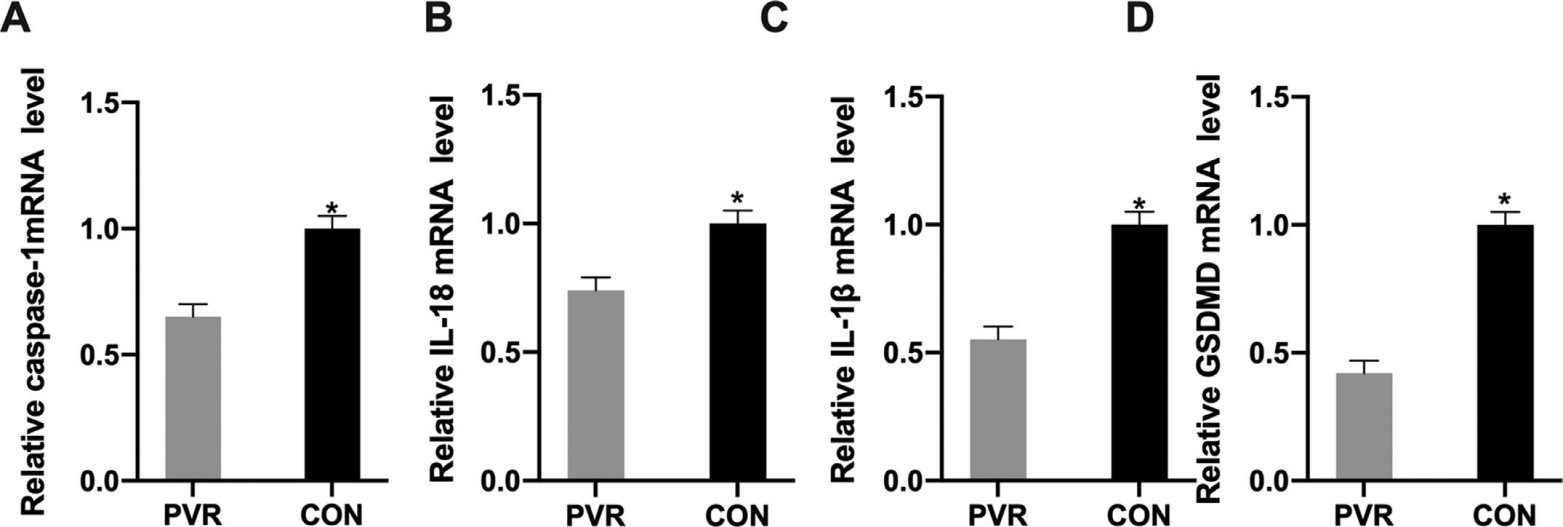
Expression of pyroptosis-related genes in PVR patients. (A) *CASP1*, (B) *IL1B*, (C) *IL18,* and (D) *GSDMD* mRNA levels in the retinal proliferative membrane tissue samples analyzed by qRT-PCR. *p < 0.05, compared with the control group. PVR, Proliferative Vitreoretinopathy; *CASP1*, Cysteinyl Aspartate Specific Proteinase; *IL1B*, Interleukin-1β; *IL18*, Interleukin-18.

The authors collected retinal proliferative membrane and the surrounding normal tissue samples from 20 PVR patients in accordance with the strict inclusion and exclusion criteria. The authors analyzed the expression of apoptosis-related genes using qRT-PCR and found that, compared with the surrounding normal tissues, Pyroptosis-Related Caspase 1 (*CASP1*), *IL1B, IL18*, and *GSDMD* mRNA levels in the retinal proliferative membranes of the patients were reduced (Fig. 1).

### Expression of pyroptosis-related proteins in PVR patients

**Fig. 2:**
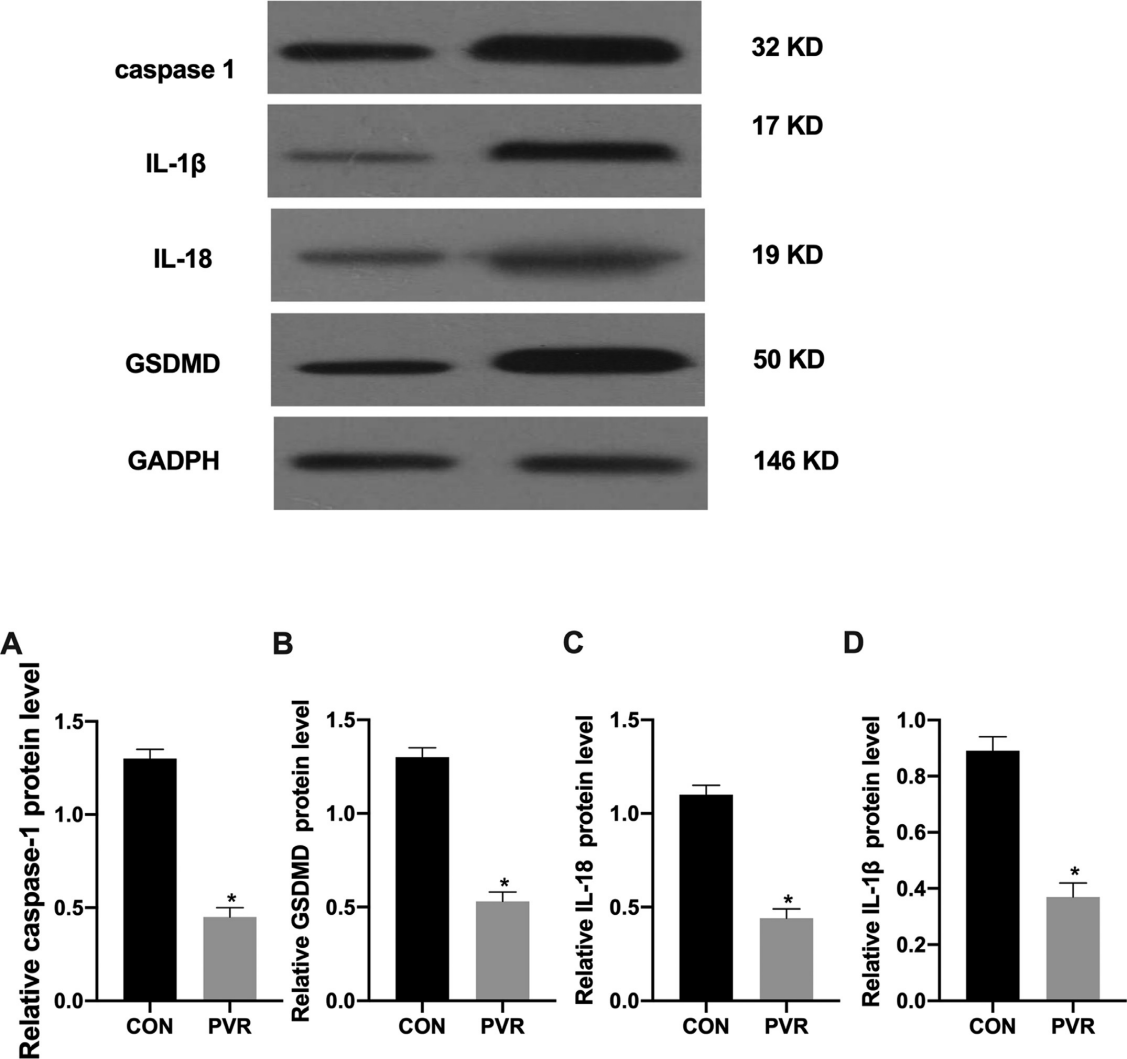
Expression of pyroptosis-related proteins in PVR patients. The expression of (A) caspase-1, (B) IL-1β, (C) IL-18, and (D) GSDMD proteins in the retinal proliferative membrane tissue samples analyzed by western blotting. *p < 0.05, compared with the control group. PVR, Proliferative Vitreoretinopathy; caspase-1, Cysteinyl aspartate specific proteinase; IL-1β, Interleukin-1β; IL-18, Interleukin-18.

The authors analyzed the expression of pyroptosis-related proteins using western blotting and found that compared with the surrounding normal tissues, pyroptosis-related caspase-1, IL-1β, IL-18, and GSDMD protein expression in the retinal proliferative membranes of the patients was decreased (Fig. 2).

### GSDMD expression in each cell group after transfection

**Fig. 3:**
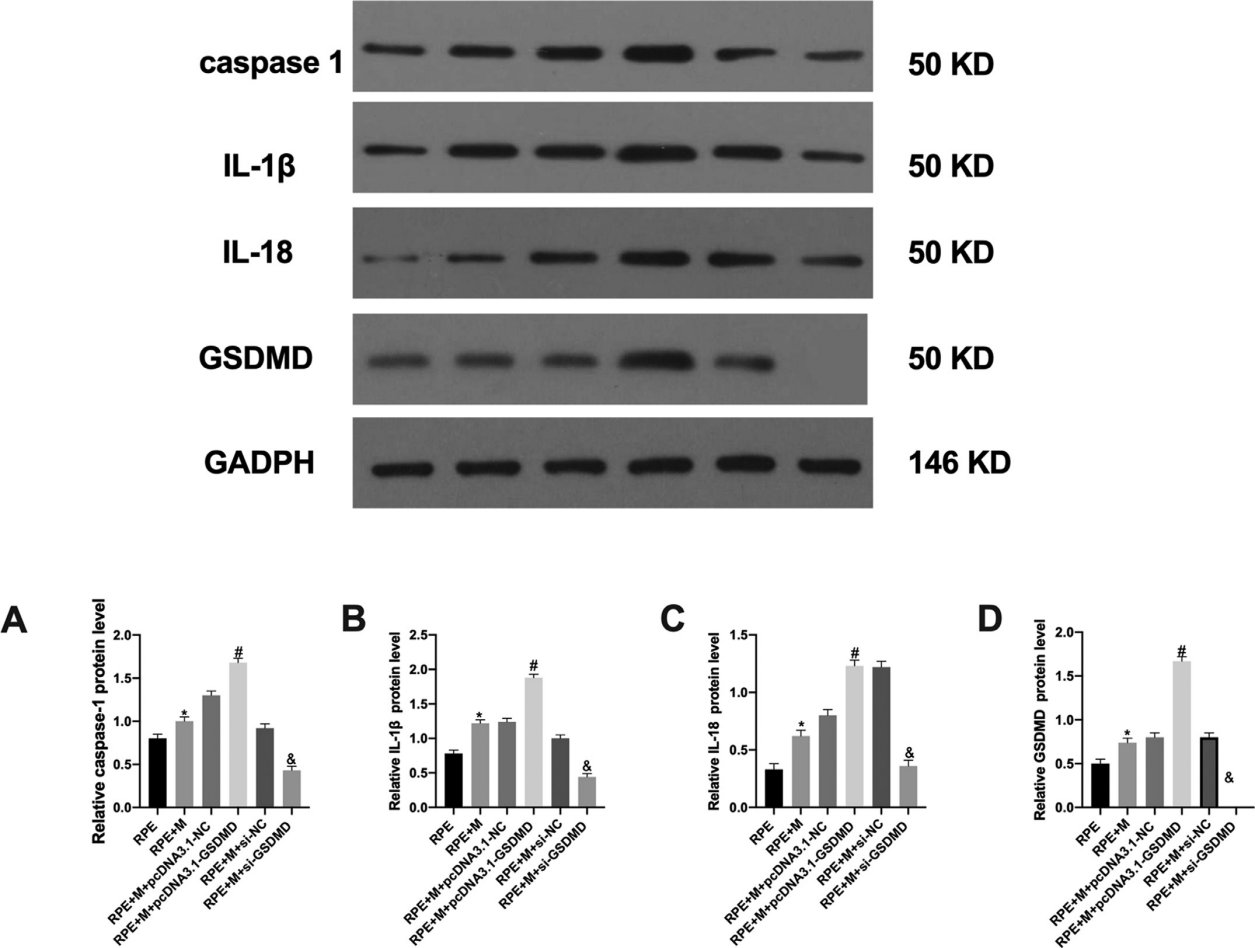
Expression of pyroptosis-related proteins in each cell group. The expression of (A) caspase-1, (B) IL-1β, (C) IL-18, and (D) GSDMD proteins in each group of cells analyzed by western blotting. *p < 0.05, compared with the control group. PVR, Proliferative Vitreoretinopathy; caspase-1, Cysteinyl aspartate specific proteinase; IL-1β, Interleukin-1β; IL-18, Interleukin-18.

The authors analyzed the expression of the pyroptosis-related GSDMD protein using Western Blot (WB) and observed that the RPE+M+pcDNA3.1-GSDMD group showed increased GSDMD expression compared with the RPE+M+pcDNA3.1-NC and RPE+M+si-NC groups. Moreover, the expression of GSDMD in the RPE+M+si-GSDMD group was reduced, indicating that the authors succeeded in constructing MC models with overexpression and downregulation of pyroptosis-related genes (Fig. 3).

### Pyroptosis-related protein expression in each cell group

The authors analyzed the expression of pyroptosis-related proteins using WB and observed that, compared with the RPE+M+pcDNA3.1-NC group, the expression of caspase-1 and IL was increased in the RPE+M+pcDNA3.1-GSDMD group. The expression of IL-1β and IL-18 increased in the RPE+M+si-NC group, while the expression of caspase-1, IL-1β, and IL-18 decreased in the RPE+M+si-GSDMD group (Fig. 3).

### MCP enhances ARPE-19 CA

**Fig. 4:**
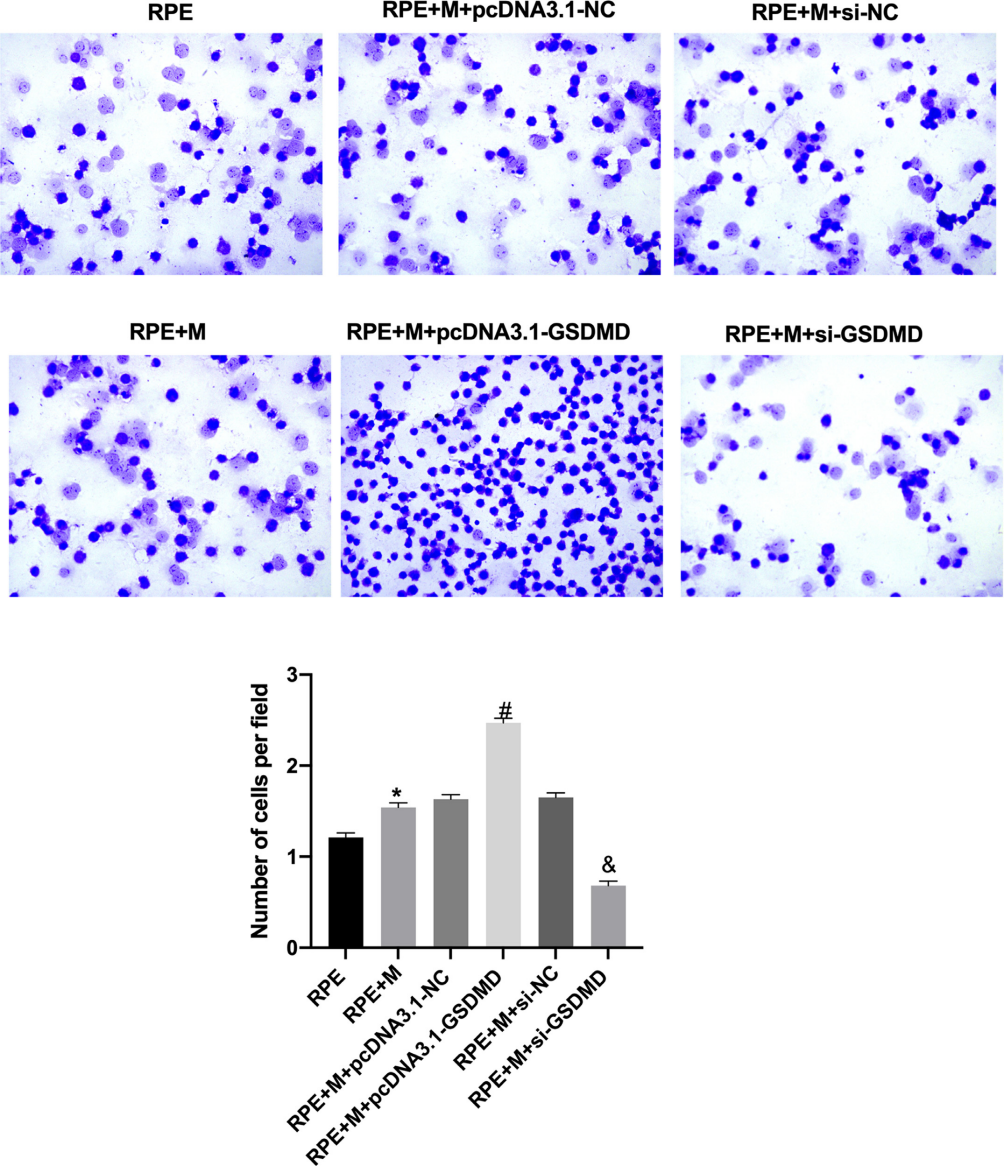
Müller cell pyroptosis enhances ARPE-19 cell adhesion. The adhesion of ARPE-19 cells in each group was analyzed using cell adhesion experiments. *p < 0.05 compared with RPE+M; ^#^p < 0.05, compared with RPE+M+pcDNA3.1; and ^&^p < 0.05, compared with RPE+M+si-NC. RPE: RPE cell-only culture group; RPE+M: RPE and Müller cell co-culture group; RPE+M+pcDNA3.1-NC: RPE and empty plasmid Müller cell co-culture group; RPE+M+pcDNA3.1-GSDMD: RPE and pyroptosis-related protein-overexpressing Müller cell co-culture group; RPE+M+si-NC: RPE and negative control siRNA Müller cell co-culture group; RPE+M+si-GSDMD: RPE and Müller cells treated with siRNA to inhibit the expression of pyroptosis-related proteins; siRNA, Small Interfering RNA.

Through CA experiments, the authors tested the adhesion of ARPE-19 cells in each group. The experimental results showed that compared with the RPE group, CA in the RPE+M group was significantly enhanced (p < 0.05). In addition, compared with the RPE+M and RPE groups, CA in the MC GSDMD overexpression group was significantly enhanced (p < 0.05). However, compared with the +M group, CA in the MCP inhibition group was significantly reduced (p < 0.05). Therefore, the authors concluded that MCP can enhance ARPE-19 CA (Fig. 4).

### MCP promotes ARPE-19 cell migration and invasion

**Fig. 5:**
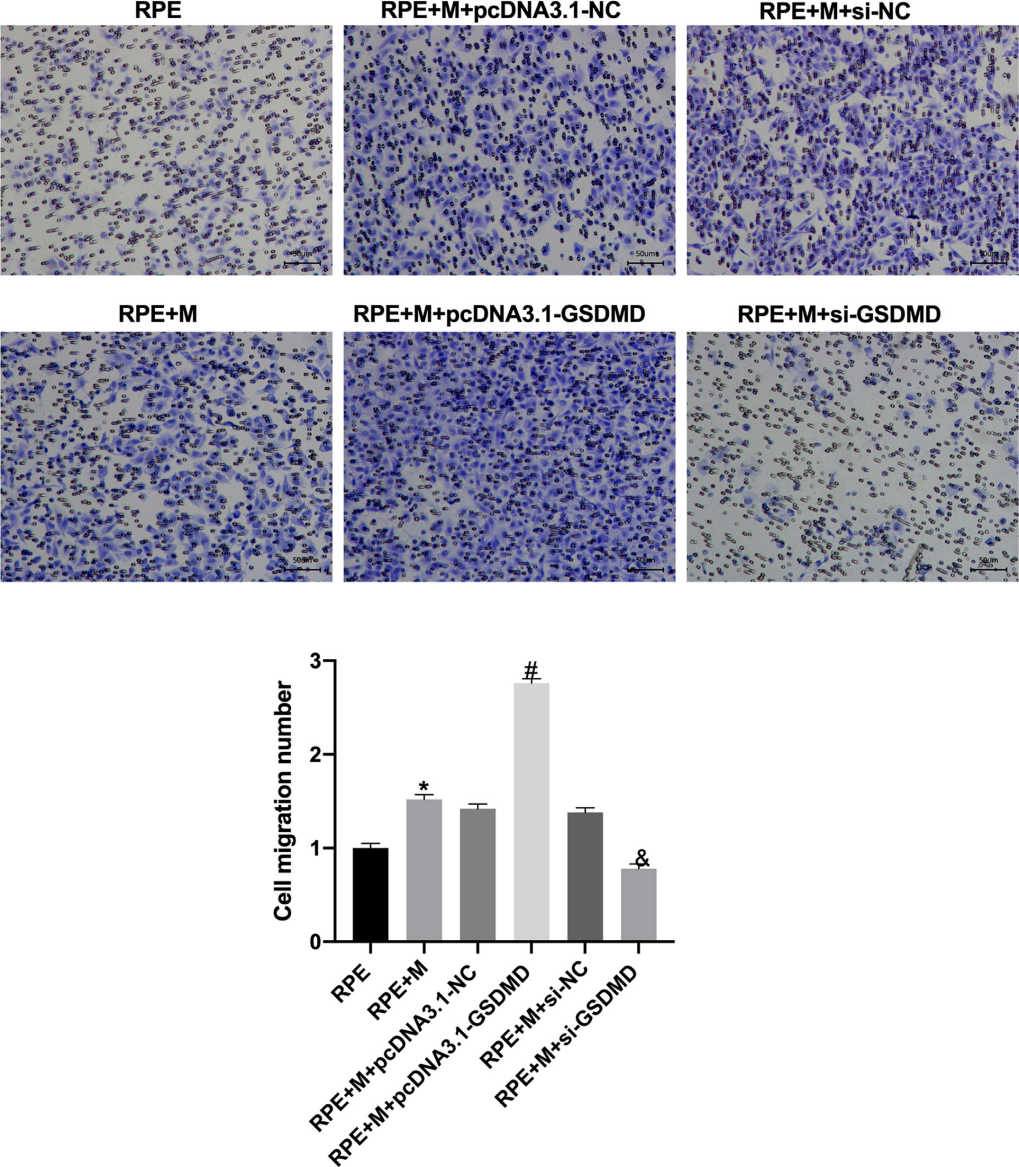
Müller cell pyroptosis promotes ARPE-19 cell migration. The Transwell assay measures the migration of ARPE-19 cells. All experiments were repeated three times, yielding the same results. *p < 0.05 compared with RPE+M; ^#^p < 0.05, compared with RPE+M+pcDNA3.1; and ^&^p < 0.05, compared with RPE+M+si-NC. RPE: RPE cell-only culture group; RPE+M: RPE and Müller cell co-culture group; RPE+M+pcDNA3.1-NC: RPE and empty plasmid Müller cell co-culture group; RPE+M+pcDNA3.1-GSDMD: RPE and pyroptosis-related protein-overexpressing Müller cell co-culture group; RPE+M+si-NC: RPE and negative control siRNA Müller cell co-culture group; RPE+M+si-GSDMD: RPE and Müller cells treated with siRNA to inhibit the expression of pyroptosis-related proteins; siRNA, small interfering RNA.

**Fig. 6:**
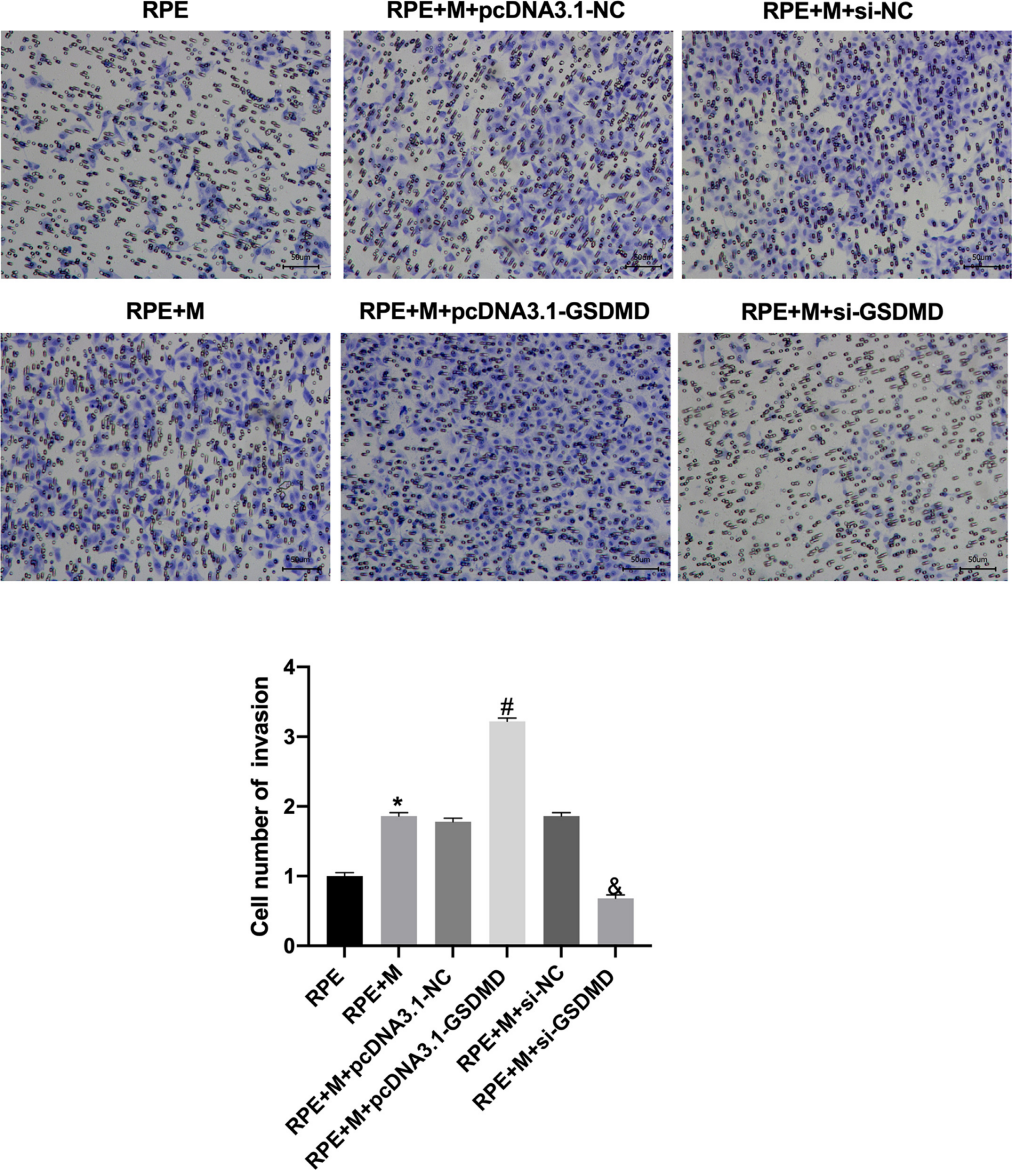
Müller cell pyroptosis promotes ARPE-19 cell invasion. The Transwell assay measures the invasion of ARPE-19 cells. All experiments were repeated three times, yielding the same results. *p < 0.05, compared with RPE+M; ^#^p < 0.05, compared with RPE+M+pcDNA3.1; and ^&^p < 0.05, compared with RPE+M+si-NC. RPE: RPE cell-only culture group; RPE+M: RPE and Müller cell co-culture group; RPE+M+pcDNA3.1-NC: RPE and empty plasmid Müller cell co-culture group; RPE+M+pcDNA3.1-GSDMD: RPE and pyroptosis-related protein-overexpressing Müller cell co-culture group; RPE+M+si-NC: RPE and negative control siRNA Müller cell co-culture group; RPE+M+si-GSDMD: RPE and Müller cells treated with siRNA to inhibit the expression of pyroptosis-related proteins; siRNA, small interfering RNA.

Through Transwell assays, the authors tested the migration and invasion capabilities of ARPE-19 cells in each group. The experimental results showed that, compared with the RPE group, the cell migration and invasion of the RPE+M group were significantly enhanced (p < 0.05). Further, compared with the RPE+M group, the cell migration and invasion of the MC GSDMD overexpression group were significantly enhanced (p < 0.05). However, compared with the RPE + M group, the cell migration and invasion of the MCP inhibition group were significantly reduced (p < 0.05). Therefore, the authors concluded that MCP can enhance ARPE-19 cell migration and invasion abilities (Fig. 5, Fig. 6).

### MCP promotes ARPE-19 cell proliferation

**Fig. 7:**
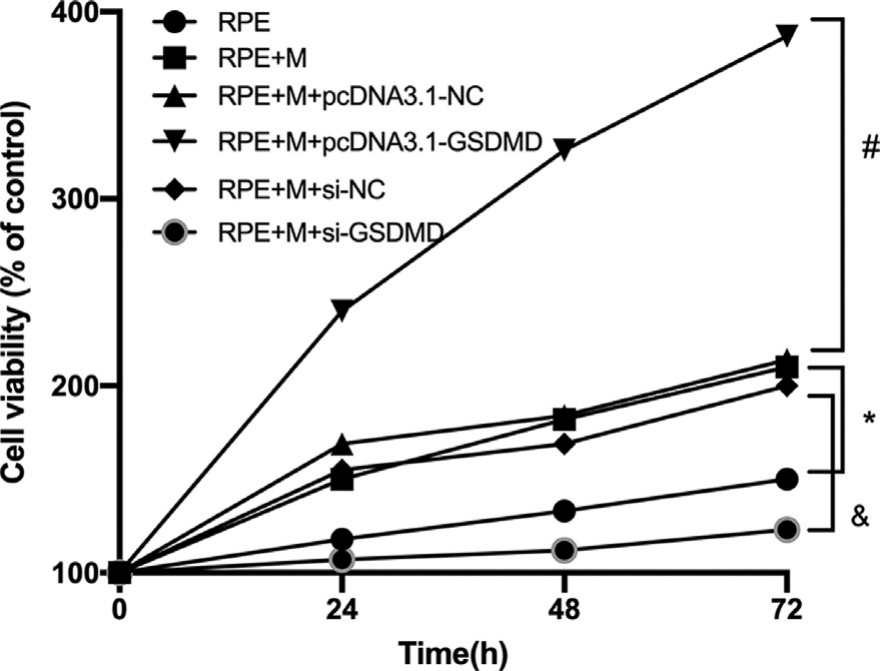
Müller cell pyroptosis promotes the proliferation of ARPE-19 cells. The CCK-8 assay measures the proliferation of ARPE-19 cells. All experiments were repeated three times, yielding the same results. * p < 0.05 compared with RPE+M; ^#^ p < 0.05, compared with RPE+M+pcDNA3.1; and ^&^ p < 0.05, compared with RPE+M+si-NC. RPE: RPE cell-only culture group; RPE+M: RPE and Müller cell co-culture group; RPE+M+pcDNA3.1-NC: RPE and empty plasmid Müller cell co-culture group; RPE+M+pcDNA3.1-GSDMD: RPE and pyroptosis-related protein-overexpressing Müller cell co-culture group; RPE+M+si-NC: RPE and negative control siRNA Müller cell co-culture group; RPE+M+si-GSDMD: RPE and Müller cells treated with siRNA to inhibit the expression of pyroptosis-related proteins; siRNA: small interfering RNA.

Using the CCK-8 assay, the authors tested the Proliferation Ability (PA) of ARPE-19 cells in each group. The experimental results showed that compared with the RPE group, the cell PA of the RPE+M group was significantly enhanced (p < 0.05). Further, compared with the RPE + M group, the cell PA of the MC GSDMD overexpression group was also significantly enhanced (p < 0.05). However, compared with the RPE + M group, the cell PA of the MCP inhibition group was significantly decreased (p < 0.05). Therefore, the authors concluded that MCP can enhance the PA of ARPE-19 cells (Fig. 7).

### MCP increases cytokine expression in ARPE-19 cells

**Fig. 8:**
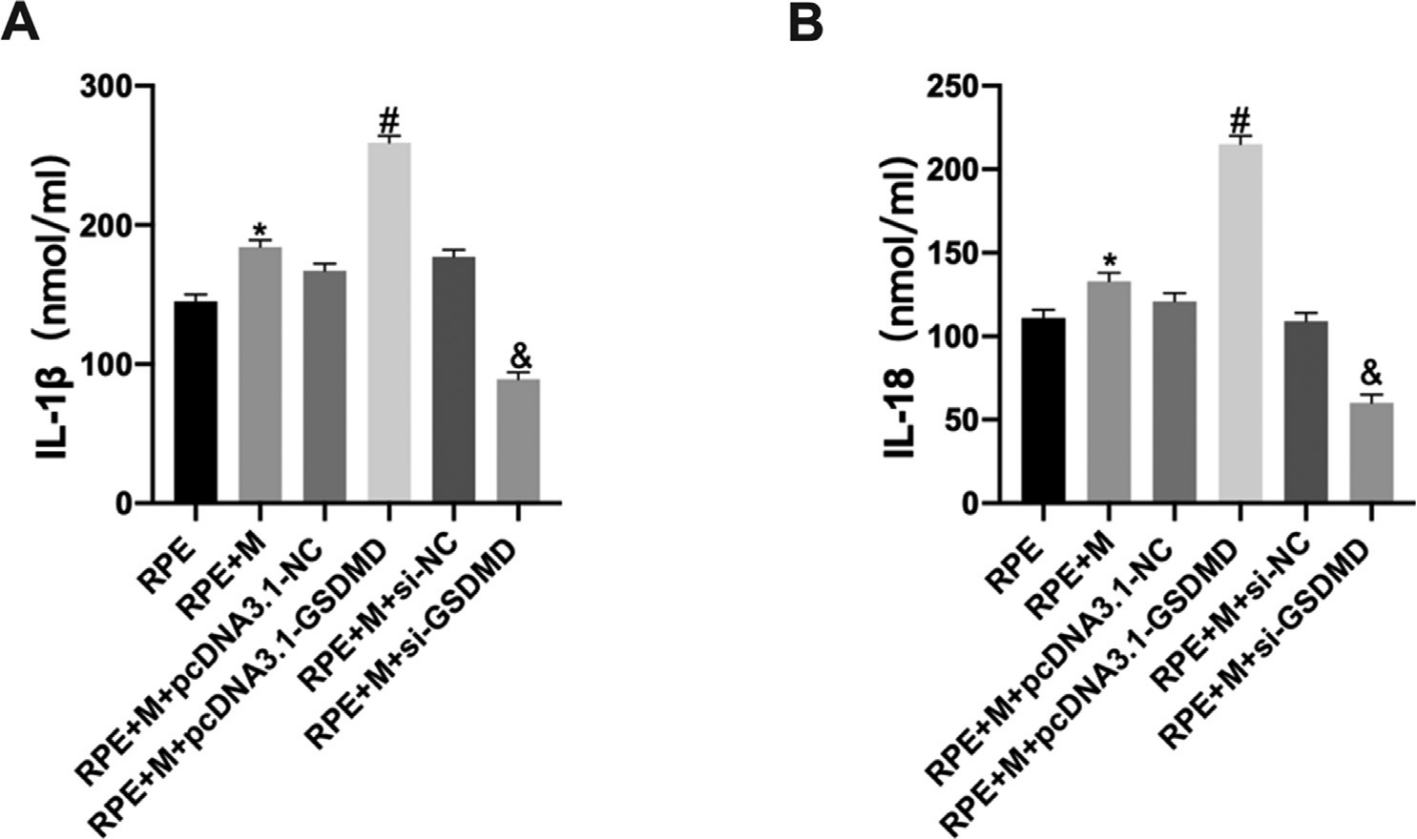
Müller cell pyroptosis promotes ARPE-19 cell cytokine expression. Cytokine expression in ARPE-19 cells was detected by ELISA. Expression of the cytokines (A) IL-1β and (B) IL-18. *p < 0.05 compared with RPE+M; ^#^p < 0.05, compared with RPE+M+pcDNA3.1; and ^&^p < 0.05, compared with RPE+M+si-NC; RPE: RPE cell-only culture group; RPE+M: RPE and Müller cell co-culture group; RPE+M+pcDNA3.1-NC: RPE and empty plasmid Müller cell co-culture group; RPE+M+pcDNA3.1-GSDMD: RPE and pyroptosis-related protein-overexpressing Müller cell co-culture group; RPE+M+si-NC: RPE and negative control siRNA Müller cell co-culture group; RPE+M+si-GSDMD: RPE and Müller cells treated with siRNA to inhibit the expression of pyroptosis-related proteins; siRNA: small interfering RNA; IL-1β: interleukin-1β; IL-18: interleukin-18.

Using ELISA, the authors detected cytokine expression in ARPE-19 cells in each group. The experimental results showed that, compared with the RPE group, the expression of IL-1β and IL-18 in the RPE+M group significantly increased (p < 0.05). Further, compared with the RPE+M group, MCs overexpressing pyroptosis-related proteins showed significantly increased expression of IL-1β and IL-18 (p < 0.05). However, compared with the RPE + M group, the expression of IL-1β and IL-18 decreased in the MCP inhibition group (p < 0.05). Therefore, the authors concluded that MCP can promote an increase in ARPE-19 cell cytokine expression (Fig. 8).

## Discussion

This study explored the role of MCP in ARPE-19 cells, and results showed that, compared with the surrounding normal tissues, the expression of caspase-1, IL-1β, IL-18, and GSDMD at both the protein and mRNA levels was reduced in the retinal proliferative membranes of the patients with PVR.

To further explore the effects of pyroptosis on RPE cells, the authors used plasmid transfection to inhibit or enhance pyroptosis in MCs. The experimental results showed that MCP enhanced ARPE-19 CA, migration and invasion, proliferation, and cytokine expression.

Therefore, the authors believe that MCP can promote the development of PVR lesions.

PVR is more common in rhegmatogenous retinal detachment and ocular trauma.^11^^,^^12^ This is because of the tendency of RPE cells, glial fibroblasts, and inflammatory cells to migrate to the vitreous cavity or the front and rear surfaces of the retina. There, they proliferate, transdifferentiate, synthesize extracellular matrix components such as collagen, and form a shrinkable cellular proliferation membrane in the vitreous cavity and on the inner and outer surfaces of the retina. Finally, vitreous pulling causes retinal detachment.^13^^,^^14^

Pyrocytosis or pyroptosis, is a recently elucidated mechanism underlying cell inflammatory death. It was reported for the first time in *Salmonella*-induced macrophage death. It was later discovered that pyroptosis was caused by damage-associated molecular patterns and pathogen-associated molecular patterns, which mediate the activation of caspase-1/4/5/11 and promote the release of IL-1β and IL-18, causing a strong inflammatory response in cells.^15^^,^^16^ GSDMD triggers the pyroptosis response and is a common substrate of caspase-1 and caspase-4/5/11. GSDMD consists of two conserved domains: the C-terminal inhibitory domain (21 Ku) and the N-terminal effector domain (32 Ku). The N-terminal domain is cytotoxic and can bind to lipid components and form holes in the cell membrane, while the full-length structure is not cytotoxic.^17^ GSDMD can be cleaved into C- and N-terminals by caspase-1/4/5/11, and its N-terminal is oligomerized in the cell membrane to form non-selective pores, releasing mature IL-18 and IL-1β, and inducing cell pyroptosis.^6^^,^^18^

Pyroptosis is the most recent programmed and inflammatory cell death mechanism discovered after apoptosis and necrosis, and it contributes to the occurrence and progression of many diseases. Studies have discovered the occurrence of GSDMD-dependent cell pyroptosis in a mouse model of diabetic cardiomyopathy. Inhibition of caspase-1 expression can reduce renal tubular cell pyroptosis and alleviate Diabetic Nephropathy (DN) kidney damage.^19^ In addition, related studies have found that pyroptosis plays an important role in various ophthalmological diseases. Pyroptosis has been observed in the eye cells of patients with severe forms of Proliferative Diabetic Retinopathy (PDR), such as traction retinal detachment and neovascularization.^20^ A related study on human retinal endothelial cells treated with high concentrations of glucose found that many pyroptosis-related factors, such as NLR family pyrin domain containing-3 (NLRP3), caspase-1, and IL-1β, were highly expressed in the proliferating retinal cells.^21^ Studies have found that pyroptosis has a role in the development of age-related cataracts, and inhibitors of the pyroptosis-related NLRP3 factor can effectively inhibit the disease's occurrence.^22^ Presently, no study has investigated pyroptosis in PVR. The results of the present study exhibited that the expression of the pyroptosis-related factors caspase-1, IL-1β, IL-18, and GSDMD at the protein and mRNA levels in the retinal proliferative membrane increased the incidence of PVR. Therefore, the authors speculate that cell pyroptosis occurs during PVR.

PVR is an overreaction to the active reconstruction process initiated by the injured retina. Glial cells play an important role in this process. MCs are the main glial cells of the retina and play an important role in maintaining retinal homeostasis.^23^^,^^24^ In PVR, this initiates the process of non-specific tissue repair that mainly manifests as hypertrophy of MCs. After retinal detachment, MCs migrate to the outer nuclear and outer plexiform layers within three days, occupy the dead photoreceptor cells, and expand into the subretinal space.^25^ RPE cells can become detached in the vitreous cavity following retinal damage and undergo an epithelial-mesenchymal transition to further differentiate into myofibroblasts, leading to the formation of fibrous tissue in PVR.^26^ These cells undergo hypertrophy and replace the lost retinal neurons, causing the retina to shorten.^27^

Relevant studies have shown that MCP is associated with proliferative diabetic retinopathy. Stienstra et al. reported that MCs exhibit increased caspase-1 activity and IL-1β production after exposure to hyperglycemic conditions, which causes the cells to die.^28^ An earlier study analyzed MCs in the retinas of healthy and diabetic rats and found that the mortality of MCs in the retinas of diabetic rats was significantly higher.^29^ The same outcome was obtained when the authors used the caspase-1/IL-1β pathway to prevent diabetes-induced MC death *in vivo*. Therefore, MCP plays an important role in proliferative diabetic retinopathy. To further explore the role of MCP in PVR, the authors conducted *in vitro* experiments. The results showed that MCP enhanced ARPE-19 CA, migration and invasion, PA, and cytokine expression. These findings indicate that the inhibition of MCP may be the key to the treatment of PVR.

IL-18 is a pleiotropic pro-inflammatory cytokine. It plays an important regulatory role in the inflammatory response and participates in the pathological process after trauma. According to certain studies, the level of IL-18 in the vitreous cavity in the PVR group was significantly higher compared to the control group, reaching a peak at three to five days.^30^ IL-18 can increase the expression of Fas Ligand (FasL), thereby activating the Nuclear Factor kappa-light-chain-enhancer of activated B cells (NF-κB), the secondary inflammatory mediator, IL-1β, and other transcription factors. IL-1β, in turn, can activate the NF-κB pathway, forming a positive feedback loop and leading to excessive tissue development, inflammation, and injury.^31^ The results of the present study showed that inhibiting pyroptosis in MCs could significantly reduce the secretion of IL-1β and IL-8 in ARPE-19 cells, thereby decreasing the further occurrence of MCP. Therefore, MCs prevent the further progression of PVR by inhibiting pyroptosis and reducing the secretion of the inflammatory factors IL-1β and IL-8 in ARPE-19 cells.

This study has some limitations. First, the authors only conducted related experiments *in vitro*, and the authors did not verify the effect of MCP on RPE in animals. In addition, for the detection of MCP, due to the limitations of experimental conditions, the authors did not observe the retinal structures under an electron microscope. The authors will further improve the methodology upon securing sufficient experimental funds at a later stage.

## Conclusions

In conclusion, the present study found that pyroptosis occurs in PVR. MCP can enhance ARPE-19 CA, proliferation, migration, and invasion ability; increase the secretion of the inflammatory factors IL-1β and IL-8; and promote the further development of PVR. These results provide new insights into PVR treatment.

## Authors’ contributions

All authors contributed to the study's conception and design. Material preparation, data collection, and analysis were performed by Yue Bai and Maosong Xie. The first draft of the manuscript was written by Yue Bai. Yihua Zhu commented on previous versions of the manuscript. All authors read and approved the final manuscript.

## Declaration of Competing Interest

The authors declare no conflicts of interest.
